# How stressful was the COVID-19 vaccination procedure? Comparison between mass vaccination centers and general practices

**DOI:** 10.1016/j.jvacx.2024.100524

**Published:** 2024-07-09

**Authors:** Anne Schrimpf, Anne Jentzsch, Markus Bleckwenn, Anne-Kathrin Geier

**Affiliations:** Institute for General Practice, Faculty of Medicine, Leipzig University, Germany

**Keywords:** COVID-19, Vaccines, Immunization programs, Preventive health services, Primary health care, Mass vaccination, Quality of health care, Needs assessment

## Abstract

Negative past experiences with vaccines or unfamiliar environments can be sources of stress during the COVID-19 vaccination procedure. We examined whether the perceived stressfulness of the vaccination procedure differ between mass vaccination centers and general practitioner (GP) practices. A survey was distributed (07/2021–10/2021) among newly vaccinated individuals in ten GP practices (n = 364) and two vaccine centers (n = 474). Stress was low at all sites. The perceived stressfulness of the procedure was higher among younger participants and those in GP practices, and increased with longer waiting time at the site. Stress decreased with better comprehensibility of the procedure and higher satisfaction with patient education. Participants who expressed greater concern about the health risks of COVID-19 vaccines perceived the vaccination procedure as more stressful. Our findings indicate opportunities for improvements in future vaccination campaigns and highlight the important role of healthcare providers in mitigating stress by addressing individual concerns.

## Introduction

Vaccination is an effective tool for preventing and controlling infectious diseases. In response to the COVID-19 pandemic, several vaccines were rapidly developed worldwide. In Germany, authorized vaccines were first administered in mass vaccination centers in December 2020. General practitioners (GPs) joined the national vaccination strategy in April 2021 [1], [2].

The vaccination procedure can be a source of stress for some individuals, particularly those who have had negative experiences with vaccines in the past or are less familiar with the process or the environment. For example, the procedures in unfamiliar vaccination centers – converted public spaces designed to vaccinate as many people as possible per day – could be confusing and therefore a potential stressor [3]. Negative past experiences with vaccines [4], fear of side effects [5], needle phobia [6], or anxiety [7] can also contribute to stress prior to or during vaccinations. For these reasons, especially older individuals might feel more comfortable in their usual GP practice [8].

Reducing stressful experiences during vaccination procedures is therefore not only important to increase the likelihood of future vaccination intentions, but also increases vaccination efficacy, as long-term (e.g., chronic anxiety or depression) and short-term (e.g., exams) stress has been shown to impair the antibody response (see review by [9]). Further, pre-vaccination fear was associated with increased incidences of adverse reactions after vaccination [10]. Healthcare providers can therefore play a crucial role in addressing these issues and reducing stress by providing education, reassurance, and support to individuals during the vaccination procedure.

Our study aimed to assess and compare the perceived stressfulness of the vaccination procedure – including arrival and registration, waiting, vaccination education and health check by the physician, vaccine administration, and observation period – among newly vaccinated individuals by administering a paper-based survey. Specifically, we sought to investigate whether there were significant differences in the perceived stress levels between those who received vaccinations at GP practices versus those at mass vaccination centers. Given the potential familiarity with the GP practice and its staff due to prior interactions, as well as the potential provision of better individual patient education due to GPs' knowledge of their patients' medical histories, we hypothesized that the procedure in GP practices might be less stressful compared to mass vaccination centers. This effect may be particularly pronounced among older individuals. We further explored if waiting time prior to and perception of stay after receiving the vaccine, comprehensibility of the vaccination procedure, satisfaction with patient education, societal expectations, or health risk concerns might additionally influence the stressfulness of the procedure.

## Methods

### Sampling and design

The data were collected between 07/2021 and 10/2021 in the Free State of Saxony, Germany. Vaccinated individuals older than 18 years of age, who had scheduled their vaccination appointments in advance at either site, completed the cross-sectional questionnaire immediately after receiving their first or second COVID-19 vaccine within their recommended 15 min observation time. The questionnaires were distributed in vaccination centers (city area of Leipzig/rural area of Belgern) and GP practices in city and in rural areas (city area of Leipzig/ rural area, Leipziger Land/Nordsachsen). The two participating vaccination centers received in total n = 650 questionnaires (n = 350 in the city area and n = 300 in the rural area). Ten GP practices received in total n = 450 questionnaires, of which five GP practices were located in the city area (n = 195 questionnaires) and five practices were located in a rural area (n = 255 questionnaires). More details on the recruitment procedure were reported elsewhere [11].

### Questionnaire

The questionnaire was self-developed and underwent a think-aloud pre-testing. It was adapted to the respective vaccination site in terms of wording and included the following topics: 1) socio-demographics, 2) reason for vaccination, 3) vaccination access, 4) vaccination procedure, and 5) attitudes and perceptions regarding health risk concerns, vaccination procedure, and stressfulness of the procedure. Attitudes were assessed by means of either 5-point or 10-point rating scales. An overview of the subset of questions included in the present study can be found in Table 1.

**Table 1:** Sociodemographic sample characteristics and variables included in this study.

	Total	Vaccine center city	Vaccine center rural	GP practices city	GP practices rural
n	838	246	228	169	195
Age	42.5 ± 16.1	36.2 ± 14.1	40.5 ± 14.4	48.5 ± 17.6	48.1 ± 15.6
Gender					
Female	406 (51.4)	119 (50.0)	107 (50.2)	93 (57.8)	87 (48.9)
Male	380 (48.1)	117 (49.2)	104 (48.8)	68 (42.2)	91 (51.1)
Diverse	4 (0.5)	2 (0.8)	2 (0.9)	0 (0.0)	0 (0.0)
Education					
Primary	10 (1.2)	1 (0.4)	3 (1.3)	2 (1.2)	4 (2.2)
Secondary	560 (68.4)	143(58.4)	159 (70.7)	130 (78.8)	128 (69.6)
Tertiary	249 (30.4)	101 (41.2)	63 (28.0)	33 (20.0)	52 (28.3)
Wait time at vaccine site					
<10 min	565 (70.4)	200 (83.7)	178 (80.5)	98 (62.4)	89 (47.8)
10–30 min	196 (24.4)	36 (15.1)	36 (16.3)	50 (31.8)	74 (39.8)
31–60 min	36 (4.5)	2 (0.8)	6 (2.7)	8 (5.1)	20 (10.8)
>60 min	6 (0.7)	1 (0.4)	1 (0.5)	1 (0.6)	3 (1.6)
**Reasons for vaccination:** Societal expectations.					
No	767 (91.5)	218 (88.6)	205 (89.9)	160 (94.7)	184 (94.4)
Yes	71 (8.5)	28 (11.4)	23 (10.1)	9 (5.3)	11 (5.6)
**Health risk concerns**: I had concerns about the health risks of COVID-19 vaccines.					
1 = Strongly disagree 5 = Strongly agree	2.56 ± 1.31	2.55 ± 1.24	2.52 ± 1.33	2.78 ± 1.34	2.45 ± 1.33
**Comprehensibility of the vaccination procedure**: The vaccination procedure was easy to understand.					
1 = Strongly disagree 5 = Strongly agree	4.67 ± 0.89	4.63 ± 0.84	4.66 ± 0.95	4.69 ± 0.87	4.70 ± 0.88
**Satisfaction with patient education**: How satisfied were you with the vaccination education?					
1 = Not at all satisfied	8.83 ± 1.91	8.52 ± 2.03	9.01 ± 1.69	8.51 ± 2.22	9.31 ± 1.58
10 = Very satisfied					
**Perception of stay**: How did you perceive your stay at the GP practice/vaccine center after the vaccination?					
1 = Not at all comfortable	8.87 ± 1.64	8.54 ± 1.70	9.00 ± 1.55	9.01 ± 1.63	9.03 ± 1.62
10 = Very comfortable					
**Stressfulness**: How stressful did you perceive the vaccination procedure?					
1 = Not stressful at all	1.91 ± 1.85	2.01 ± 1.80	1.75 ± 1.81	2.22 ± 2.20	1.71 ± 1.57
10 = Very stressful					

*Note.* Values represent mean and standard deviation or n and percentage of valid cases (%), respectively.

### Ethics statement

The study was carried out in accordance with the Declaration of Helsinki. The study protocol was approved by the research ethics committee of the Leipzig University (reference number 314/21-ek). Participants agreed to participate by voluntarily returning the anonymous questionnaire. No personal data besides age and sex were assessed.

### Statistical analyses

All statistical analyses were carried out using IBM SPSS Statistics 27 (Armonk, NY, USA). For descriptive statistics, missing values in single variables were considered by presenting frequencies as % (n/n_valid_). Continuous variables were presented as mean ± standard deviation (SD). As the continuous variable “stressfulness” was not normally distributed, we analyzed univariate location differences (“vaccine center city”, “vaccine center rural”, “GP practices city”, “GP practices rural”) by using a Kruskal‐Wallis test and subsequent post-hoc tests with Bonferroni corrections. Estimated effect sizes are reported using Cohen’s *d*.

Further, a multiple linear regression analysis using Entry method was conducted. The predictive ability of demographic variables, waiting time at site, reasons for vaccination, comprehensibility of the vaccination procedure, satisfaction with patient education, perception of stay after vaccination, and health risk concerns (independent variables) in explaining stressfulness of vaccination procedure (dependent variable) were calculated.

## Results

### Sample characteristics

A total of 1100 questionnaires were distributed to two vaccine centers and ten primary care practices, of which 838 (GP practices n = 364, vaccination centers n = 474) were completed and eligible for analysis (response rate of 76.2 %). Further information on sample characteristics were reported elsewhere [11] and in Table 1.

### Perceived stressfulness of the vaccination procedure between sites

We examined the stressfulness of the vaccination procedure on a 10-point rating scale. The analyses showed that the perceived stressfulness differed between sites (*H*(3) = 13.245, *p* = 0.004, *d* = 0.23). Considering a general low level of stress at all sites and a low effect size, the post-hoc results showed that only two pairs remained significant: GP practices rural and vaccine center city (z = 2.895, p = 0.023, *d* = 0.28) and vaccine center city and vaccine center rural (z = -2.921, p = 0.021, *d* = 0.27; see descriptives in Table 1).

### Predicting stressfulness of vaccination procedure

A multiple linear regression was calculated to predict stressfulness of the vaccination procedure based on waiting time at site, reasons for vaccination, comprehensibility of the vaccination procedure, satisfaction with patient education, perception of stay after vaccination, health risk concerns, and demographic variables. The model explained 22.7 % of the variation in stressfulness of the vaccination procedure (*F*(10,675) = 19.878, *p* < 0.001; Table 2).

**Table 2:** Multiple regression analysis predicting stressfulness of the vaccination procedure.

Predictor	*B*	*SE B*	β	*R*^2^
Stressfulness of the vaccination procedure				
				0.227
Constant	5.189	0.657		
Age	−0.010	0.004	−0.088*	
Education	0.057	0.119	0.017	
Vaccination center/GP practice	0.316	0.134	0.087*	
City/rural	0.239	0.124	0.067	
Waiting time at vaccine site	0.419	0.109	0.138**	
Reasons for vaccination: Societal expectations	0.538	0.219	0.084*	
Comprehensibility of the vaccination procedure	−0.188	0.078	−0.084*	
Satisfaction with patient education	−0.115	0.038	−0.120*	
Perception of the stay after vaccination	−0.318	0.045	−0.279**	
Health risk concerns	0.123	0.048	0.089*	

*Note*. Durbin-Watson = 1.918, * p < 0.005, ** p < 0.001.

## Discussion

In this study, we compared the perceived stressfulness of the vaccination procedure between vaccinees in GP practices (n = 364) and mass vaccination centers (n = 474). In general, perceived stress was very low at all sites. Multivariate regression showed that, contrary to our hypotheses, the perceived stressfulness of the vaccination procedure was higher among younger participants and those in GP practices. Recent studies have also found that younger individuals exhibited more pre-vaccination stress than older individuals [5], [7]. One explanation could be that younger participants might have less experience with medical procedures and are less likely to receive annual vaccines, such as influenza. Additionally, side effects of COVID-19 vaccines were more prevalent in younger than in older individuals [12], which attracted media attention at the time of the study and may have enhanced stress in younger individuals during the vaccination procedure. Although interpretations are speculative, the higher perceived stress in GP practices compared to mass vaccination centers could be explained by differences in waiting time or crowding, as vaccination procedures in mass vaccination centers have been shown to be more robust to increased crowding and staff shortages [13]. Further, consultations in GP practices were not restricted to vaccinations, resulting in a variety of patients with different needs waiting in the practices. Other factors, such as vaccine administration protocols, might have contributed to the observed differences.

In support of the above made assumption, we further found that the procedure was perceived to be more stressful in case of longer waiting time at the vaccination site before vaccination and a less pleasant stay after vaccination, whereas a better comprehensibility of the vaccination procedure and higher satisfaction with patient education reduced perceived stress. As waiting times increased with staff shortages in both mass vaccination centers and GP practices [13], our results suggest that stressfulness of vaccination procedures could be reduced by sufficient staffing. Further, the link between a comprehensible vaccination procedure and patient education with reduced stress indicates that stress and uncertainty during vaccination procedures can be decreased by easy-to-understand processes, clear communication, and the provision of adequate and accessible information, which is consistent with recent research [14]. Healthcare providers therefore play a vital role in creating a supportive and reassuring environment for vaccine recipients.

Lastly, the regression revealed that participants who stated societal expectations as a reason for vaccination also perceived the vaccination procedure as more stressful. Social pressure was found to be a factor for vaccination during the COVID-19 pandemic, potentially driven by the fear of exclusion from social events or stigma [15], [16]. Pressure to conform might intensify perceived stress during the vaccination procedure. In addition, participants who expressed greater concern about the health risks of COVID-19 vaccines were also more likely to perceive the vaccination procedure as more stressful. Recently, low trust in the efficacy of COVID-19 vaccines has been shown to increase psychological distress when imagining being vaccinated [5]. It is conceivable that individuals with higher health risk concerns may be more apprehensive about and pay more attention to potential side effects or adverse reactions associated with the vaccine, leading to heightened stress during the vaccination procedure.

## Limitations

All responses were self-reports, which might be imprecise due to subjective perceptions. As the study was carried out in a single federal state in Germany, socio-demographic differences between federal states in Germany as well as between European countries could restrict the generalizability of our findings. Further, the availability and accessibility of mass vaccination centers/GPs, vaccine supply, case incidences, and vaccination willingness might have differed across Europe and other regions globally, challenging direct comparability. Additionally, another potential limitation is the possibility of selection bias. The low stress levels reported across all sites could be attributed to the reluctance of highly stressed individuals to participate in the survey. This self-selection bias might have led to an underrepresentation of stressed individuals in our sample, potentially limiting the generalizability of our findings.

## Implications

Perceived stressfulness of the vaccination procedure was low at all sites, indicating there was already a very high standard of the processes. However, it is important to further reduce stress during vaccination procedures to avoid adverse events [6], [10] and increase vaccine uptake. Future vaccination campaigns could additionally improve the vaccinees’ experience through more comprehensive procedures, shorter waiting times on site, and more comfort during the fifteen-minute post-vaccination waiting period (e.g., through separate and quiet rooms). Our results also suggest that providing additional staff, particularly in GP practices, during periods of excessive vaccination demand could further reduce stress among vaccinees. We also highlight the important role of the health care provider in reducing stress during the procedure by providing sufficient and adequate information about the vaccine, e.g., with respect to health risks or side effects and by addressing individual concerns. An inclusive environment, free from additional pressures on individual decision-making, could alleviate stress and decrease resistance in future vaccination events [15]. Our findings can help healthcare providers tailor their communication and support strategies to address concerns and mitigate stress for individuals undergoing vaccination.

## Conclusion

We examined whether the stressfulness of the vaccination procedure would differ between mass vaccination centers and GP practices. We confirm that although the perceived stressfulness of the vaccination procedure was low at all vaccination sites, stress was higher in GP practices and among younger individuals, and increased with longer waiting times and with a less pleasant post-vaccination waiting period. Stress also increased with greater perceived societal expectations to get vaccinated and with more concerns about health risks of COVID-19 vaccines. Stress decreased with a more comprehensible vaccination procedure and more satisfactory patient education, emphasizing the important role of healthcare providers in educating and reassuring patients to reduce stress during the vaccination procedure. Our findings provide insights into how future vaccination campaigns could be designed to reduce stress and to optimally meet individual needs.

## CRediT authorship contribution statement

**Anne Schrimpf:** Conceptualization, Formal analysis, Methodology, Project administration, Supervision, Writing – original draft. **Anne Jentzsch:** Conceptualization, Formal analysis, Methodology, Project administration, Supervision, Writing – original draft. **Markus Bleckwenn:** Conceptualization, Supervision, Writing – review & editing. **Anne-Kathrin Geier:** Conceptualization, Formal analysis, Methodology, Project administration, Supervision, Writing – review & editing.

## Declaration of competing interest

The authors declare that they have no known competing financial interests or personal relationships that could have appeared to influence the work reported in this paper.

## Data Availability

Data will be made available on request.
